# Individual and Population-Level Impacts of an Emerging Poxvirus Disease in a Wild Population of Great Tits

**DOI:** 10.1371/journal.pone.0048545

**Published:** 2012-11-21

**Authors:** Shelly Lachish, Michael B. Bonsall, Becki Lawson, Andrew A. Cunningham, Ben C. Sheldon

**Affiliations:** 1 Edward Grey Institute, Department of Zoology, University of Oxford, Oxford, United Kingdom; 2 Mathematical Ecology Research Group, Department of Zoology, University of Oxford, Oxford, United Kingdom; 3 Institute of Zoology, Zoological Society of London, London, United Kingdom; University of Georgia, United States of America

## Abstract

Emerging infectious diseases of wildlife can have severe effects on host populations and constitute a pressing problem for biodiversity conservation. Paridae pox is an unusually severe form of avipoxvirus infection that has recently been identified as an emerging infectious disease particularly affecting an abundant songbird, the great tit (*Parus major*), in Great Britain. In this study, we study the invasion and establishment of Paridae pox in a long-term monitored population of wild great tits to (i) quantify the impact of this novel pathogen on host fitness and (ii) determine the potential threat it poses to population persistence. We show that Paridae pox significantly reduces the reproductive output of great tits by reducing the ability of parents to fledge young successfully and rear those young to independence. Our results also suggested that pathogen transmission from diseased parents to their offspring was possible, and that disease entails severe mortality costs for affected chicks. Application of multistate mark-recapture modelling showed that Paridae pox causes significant reductions to host survival, with particularly large effects observed for juvenile survival. Using an age-structured population model, we demonstrate that Paridae pox has the potential to reduce population growth rate, primarily through negative impacts on host survival rates. However, at currently observed prevalence, significant disease-induced population decline seems unlikely, although pox prevalence may be underestimated if capture probability of diseased individuals is low. Despite this, because pox-affected model populations exhibited lower average growth rates, this emerging infectious disease has the potential to reduce the resilience of populations to other environmental factors that reduce population size.

## Introduction

Emerging infectious wildlife diseases can have severe negative effects on host populations, causing marked population declines and increasing extinction risk in a variety of taxonomic groups [1], [2], [3], [4], [5]. Moreover, epidemics of novel infectious diseases not only pose a significant extinction risk for already small and fragmented populations [6], [7], but they can also threaten the viability of common species with large geographic distributions [1], [8]. Consequently, the unprecedented recent increase in the identification of emerging wildlife diseases [9] has raised concerns that emerging pathogens may pose a substantial threat to the maintenance of global biodiversity [10], [11], [12], and has highlighted the need to rapidly determine any threats that novel pathogens pose to the persistence of wildlife populations.

The degree to which a pathogen impacts on population dynamics is governed not only by the extent of infection in the population (and in populations of any reservoir hosts), but by the magnitude of the fitness costs exerted by the pathogen and the particular vital rates affected [13], [14]. Hence, ascertaining the impact of a novel pathogen on the dynamics and persistence of its host population requires not only estimates of population prevalence, but more importantly an accurate assessment of the effect of the pathogen on host survival and reproduction in the wild. Such information is also critical for predicting population responses to disease control measures and thus for effectively managing populations whose future persistence is threatened by disease impacts [15], [16]. However, quantifying the demographic consequences of pathogen infection under natural conditions is often difficult. Directly monitoring longevity and reproductive output in diseased and healthy individuals is challenging, given the need for sizeable, long-term data sets and the significant financial and logistical constraints involved in regularly capturing and diagnosing disease in large numbers of wild animals [13]. The appearance of a novel pathogen in a long-term monitored population of wild animals, however, can provide a unique opportunity to determine accurately the impacts of a pathogen on host vital rates and assess the effect of disease on population viability [17], [18].

Paridae pox is an unusually severe form of avipoxvirus infection that has recently been identified as an emerging infectious disease affecting wild tit species, predominantly great tits (*Parus major*), in Great Britain [19]. Although the disease is considered endemic in GB in non-Paridae species (e.g. common wood-pigeon *Columba palumbus*, dunnock *Prunella modularis*), avian pox was unknown within the Paridae family in GB prior to the index case, which was observed in Sussex, England in 2006 [19]. Where avian pox is endemic, the disease typically exists at very low prevalence and is presumed to have little impact on hosts, as lesions usually are mild and affected birds frequently recover [20], [21]. However, in naïve populations and species the disease can attain much higher prevalence, and has been shown to increase host mortality rate [18] and contribute to substantial population declines [2], [22], [23], [24].

Preliminary epidemiological observations suggest that Paridae pox in GB may negatively impact on wild great tit populations. Pox lesions observed in great tits are frequently larger and more florid than the small wart-like lesions characteristic of infections in non-Paridae species, and were considered a significant contributory factor to the cause of death of birds examined post mortem [19]. Furthermore, epidemiological monitoring of the recent invasion and establishment of Paridae pox in a long-term study site where great tits have been monitored for several decades (Wytham Woods, in south-east England) [25] has revealed that this novel disease can attain unusually high local prevalence (10%) for avian pox in continental areas, and may also increase the host mortality rate (as diseased birds were less likely to be recaptured than were healthy birds) [26]. In this study, we capitalise on the opportunity provided by the establishment of this novel disease in our long-term study population to quantify the impact of Paridae pox on great tits and, subsequently, to assess the potential effects of this emerging disease on population viability. We quantified the impact of Paridae pox on great tit survival and reproductive performance using multistate mark-recapture models and generalised linear models, and subsequently determined the consequences of observed disease-associated reductions in survival and reproductive output for population growth rate using an age-structured population model.

## Methods

### Ethics Statement

This study was carried out in conformity with the UK Wildlife and Countryside Act (1981). Capture of live birds was conducted in accordance with the requirements and protocols of the British Trust for Ornithology bird ringing scheme. All field workers involved in this project possessed personal ringing licenses issued by the British Trust for Ornithology, and work in Wytham Wood was authorised by BCS. All methods were approved by the Oxford University’s Ethics Committee and conducted in accordance with Oxford University’s Local Ethical Review Procedures, overseen by the Zoology Local Ethical Review Committee.

### Field Sampling

The great tit is a small passerine bird abundant in European woodlands and gardens that takes readily to nestboxes. In the UK, great tits are resident year-round and lay eggs in spring. Individually marked great tits were monitored from May 2009 (when Paridae pox was first observed in the population) to November 2011 in Wytham Woods near Oxford, UK (51°46′N, 1°20′W), as part of several on-going ecological studies [27], [28]. This 385-ha study site is a continuous mixed semi-deciduous forest, in which c.1200 nestboxes are distributed at variable densities, and of which between 250 and 400 are used for breeding by great tits each year [28]. Individual great tits were captured at nestboxes during the breeding season (May-June; the study population is single brooded). Outside the breeding season, individuals were captured with mist nets in association with seed feeders deployed throughout the woodland. Host sex was determined based either on the presence (female) or absence (male) of a brood patch (breeding birds), or on plumage coloration (males have broad black breast stripes and glossy crowns) at other times of the year [29]. During and following the autumn moult it is only possible to age newly captured individuals, based on their plumage characteristics, as either “juveniles” (aged 0–1 yr) or “older” (aged 1+ years) [29]. As a significant proportion of individuals in this study were only captured outside the breeding season (32.1%), we restricted age effects to these two age classes. During the breeding season nestboxes were checked weekly, and at day 15 all chicks present were ringed and weighed. This provides a measure of laying, hatching, fledging, and recruitment success for all reproductive attempts.

Every captured bird was carefully examined for visual signs of skin lesions. Pox lesions were identified by the presence of swellings or proliferative skin lesions, especially around the beak and eyes, legs, and on sparsely feathered parts of the wings and body. Following the identification of Paridae pox within our study site (which we confirmed using histopathological and PCR examinations of skin lesions from two diseased great tits) [19] strict hygiene protocols were implemented to minimise anthropogenic transmission and spread of pox (see details in [26]). Although exact transmission routes for this avipoxvirus system are not known, see discussion in [19], [26], pox viruses may be transmitted via direct or indirect contact, via arthropod vectors (particularly mosquitos), and possibly also via aerosol infection [21].

Previous studies have shown very good concordance between the presence of pox-like lesions and poxvirus infection [19], [21]. Hence, we are confident that birds with lesions were infected with avian poxvirus. We recognise, however, that some infected birds may have been misclassified as healthy (those incubating the disease with no clinical lesions, or whose small lesions were overlooked; although lesions as small as 1 mm were easily observed), and thus our estimates are of the effects of disease, rather than of infection.

### Impact of Pox on Great Tit Reproductive Performance

We used generalized linear models to investigate whether reproductive performance, measured at four consecutive stages of the breeding attempt, varied according to parental disease status (PDS). As we never observed both members of a pair with pox lesions, we classified PDS as ‘diseased’ if just one member of the pair was diseased. Nests where only one parent was captured and where this bird was healthy were not included in these analyses, and as only a single diseased individual was observed in 2009, these analyses were restricted to individuals breeding in 2010 and 2011. The specific reproductive measures we considered were: (i) clutch size (‘CS’), modelled as a Poisson response with a log link; (ii) the number of young alive at day 15 (‘NYoung’), modelled as a Poisson response; (iii) fledging success, modelled either as the number of young fledged (‘NFledged’; a Poisson response, adjusted for overdispersion), or as a binomial response with a logit link, indicating whether any young fledged (‘FledgedY/N’); and (iv) the number of independent young produced, again modelled either as the number of independent young produced (‘NIndYoung’; a Poisson response, adjusted for overdispersion), or as a binomial response indicating whether parents produced any independent young (‘IndYoungY/N’). Independent offspring were those that successfully fledged and later appeared within the trapped population (essentially a measure of post-fledging survival, fidelity and recapture). Lay date (‘LD’, first day of egg-laying, standardized within years), year (2010/2011), and the local breeding density (‘Density’) were fitted as covariates in all starting models as they are known to significantly influence reproductive performance in this species [30], [31]. We also fitted clutch size as a covariate when modelling ‘NYoung’, ‘NYoung’ as a covariate when modelling ‘NFledged’, and ‘NFledged’ as a covariate when modelling ‘NIndYoung’; hence each analysis addresses the additive effect of disease status at that reproductive stage, controlling for any influence on preceding stages. In addition, all models initially included several biologically meaningful interactions between the main factors (LD*year, PDS*year, PDS*Density). These models were simplified by stepwise elimination, sequentially removing terms for which P>0.1 and finally those with P>0.05 to leave the minimal model. We used a non-parametric Mann-Whitney test to investigate whether PDS influenced average brood weight (the average weight of all day 15 chicks), as the distribution of brood weights was highly left-skewed. Statistical analyses were run in R [32].

### Impact of Pox on Great Tit Survival

Multi-state mark-recapture methods were employed to obtain a robust assessment of the effect of Paridae pox on the survival rates of great tits, while accounting both for imperfect detectability and disease-dependent variation in the capture probabilities of marked individuals. The mark–recapture dataset consisted of observation histories for individuals that were captured during eight extended sampling periods from May 2009 to November 2011 (see Table S1) as these time periods were consistently sampled in multiple years, and including additional sampling periods caused lack of fit of the general multistate model due to the presence of transience and trap-dependence.

Capture histories of individuals were assigned to diseased (*D*) or healthy (*H*) states based on disease status at time of capture. We used the program UCARE v2.3.2 [33] to assess the fit of the most general model (with time and state-dependent parameters) to the data and to estimate the variance-inflation factor (*ĉ*). There was no evidence for significant lack of fit of this general model (χ^2^ = 22.80, df = 19, P = 0.25) and only slight overdispersion (*ĉ* = 1.20). However, our data were too sparse to obtain robust estimates for many of the parameters associated with the diseased state and with transitions from diseased to healthy in this fully time-structured model. We therefore simplified this general model by retaining time-dependence (*t*) only for the recapture/survival rates of healthy (*H*) individuals, keeping recapture/survival rates of diseased (*D*) individuals constant, and by retaining time-dependence (*t*) only for transitions from the healthy to diseased state (Infection: ‘*Inf*’’), keeping transitions from diseased to healthy (Recovery: ‘*Rec*’) constant. This reduced model was much better supported by the data (see Results and Table 1 for model notation) and became our starting model for subsequent model selection. Note that we subsequently evaluated two additional surrogates for temporal variation in recapture probabilities of both healthy and diseased individuals, trapping method and trapping effort (i.e. the number of trapping days per capture occasion), but neither provided a better fit to the data.

**Table 1:** Notation used to denote the main effects and model structure for multistate mark-recapture models.

Notation	Description	Parameters
State	State-dependent effect (parameters vary between healthy and diseased states, and covariates are appliedto both states)	*p*, φ, ψ
H	Healthy state (covariates apply only to this state)	*p*, φ
D	Diseased state (covariates apply only to this state)	*p*, φ
Inf	Infection transition (transition from healthy to diseased, covariates apply only to this transition)	Ψ
Rec	Recovery transition (transition from diseased to healthy, covariates apply only to this transition)	Ψ
T	Time dependence (monthly variation in parameters)	*p*, φ, ψ
Effort	Trapping effort (no. trapping days per capture session)	*p*
Sex	Sex effect (males differ to females)	φ
Age	Age effect (juveniles differ to older individuals)	φ
prev1	Pox prevalence (at the previous occasion)	φ
prev2	Pox prevalence (averaged over the preceding interval)	φ

All models were fitted to the data using program E-SURGE [34]. To ensure convergence of models on the global minima, models were run using repeated random initial values (‘multiple random’ option with N = 8; [35]). Model selection was based on small sample size corrected Akaike Information Criteria adjusted for overdispersion, QAICc [36]. Normalized QAICc weights (*w_i_*) were used as a measure of relative support for each model. We obtained robust parameter estimates through model averaging [36].

In this analysis, our primary interest was to quantify the difference in monthly survival rates of diseased and healthy individuals. Nonetheless, we employed a three-step model selection process in order to first account for variation in recapture rate (i.e. the probability that if alive and captured at time *t*, that the individual will be encountered at time *t+1*) and later explore variation in transition rate (i.e. the probability that an animal in state *a* at time *t* is in state *b* at time *t+1,* given that the animal is alive at *t+1*) among the healthy and diseased states. Hence in step 1, we modelled variation in recapture rate (of the healthy state) as state-dependent (either with or without temporal variation in the healthy state), or as time-dependent with no state effect. In step 2, we used the best recapture rate model (model with the lowest QAICc) to model survival rates in relation to disease state, time (restricted to the healthy state only), host age (juveniles, and older individuals, as defined above), and host sex (restricted to healthy state only as previous analyses have shown that prevalence and risk of disease does not vary between males and females) [26]. Note that juveniles of each year are only included in this analysis if they have fledged and survived long enough to be captured as an independent offspring (i.e. during the winter capture sessions). Hence, juvenile survival rates obtained from this approach are a measure of the survival rate of newly independent young to their first breeding season. We investigated the additive and interactive effects of these variables (to two-way interactions). Finally, in step 3 we used the best recapture and survival models identified in the previous two steps to model variation in transition rates. We modelled variation in infection rates as time-dependent, as a function of the prevalence of pox at the previous occasion (‘*prev1*’), as a function of the average prevalence over the preceding interval (‘*prev2*’), or as time invariant. In all the above models, the initial capture probability, which estimates the probability of being in a given state when first encountered [37] was allowed to vary freely with time. Model notation is given in Table 1.

### Impact of Pox on Great Tit Population Growth Rate

We used an age-structured matrix population model to translate Paridae pox impacts on great tit vital rates to disease effects on population growth rate. The population growth rate, lambda (λ), was given by the dominant eigenvalue of the transition matrix for a female-only model constructed using six age classes (juveniles, 1–2 year olds, 2–3 year olds, 3–4 year olds, 4–5 year olds, 5+year olds), an annual birth-pulse, and a post-breeding census [38]. As we were principally concerned with determining the potential extent of pox impact on population growth rate, we accepted the simplifying assumptions of density-independent growth and a closed system (though neither assumption is strictly valid for this population).

Age-specific fertility rates for healthy females (the number of females fledged per female, assuming equal sex ratios at birth) were obtained from estimates given in Bouwhuis et al. [31], which had been calculated for female great tits in this population from 1960 to 2008. Age-specific survival rates for healthy female great tits aged 1 year and older were obtained from estimates given in Bouwhuis et al., [39], calculated for the same 48 year dataset. Survival and fertility estimates for females aged five to nine years in Bouwhuis et al., [31], [39] were averaged to obtain the estimates used for the 5+age-class in our age-structured model (see Table S2 for vital rate estimates used in age-structured matrix models). Estimates of juvenile survival in great tits (calculated as local recruitment of fledglings to the breeding population) are known to be highly variable between years [40], [41], [42], [43], [44]. Large variation in juvenile survival was also evident in our study population (e.g. recruitment rate of fledglings at Wytham Woods ranged from 0.05 to 0.23 for the period from 1988 to 2008; mean = 0.12). As variation in juvenile survival rates can greatly influence population trajectories we chose to incorporate variability in juvenile survival into our estimates of λ. To do this we determined population growth rate as the average value of λ obtained after 10000 matrix model runs, in which the value for juvenile survival in the age-structured matrix at each run was drawn from a normal distribution (mean = 0.12, SD = 0.03), with draws of a negative value being discarded.

To obtain vital-rate parameters for diseased animals, we adjusted these parameters for healthy individuals according to the estimates of disease impacts detailed in this study. Fertility estimates for all age-classes were reduced by 10.5% (equivalent to the proportional reduction in the average number of young fledged by diseased parents compared with healthy parents, see Results and Table S2). Survival rates meanwhile were reduced by 26.5% per month for juveniles and 13.1% per month for all other age-classes (based on the model-averaged reduction in monthly survival rates, see Results and Table S2). Note that this will be an approximate (and probably conservative) estimate of the impact of disease on juvenile survival (i.e. survival to age one). This is because reducing juvenile survival in this way assumes that the survival costs of disease for independent young (i.e. the 26.5% reduction in average monthly juvenile survival revealed by the multistate analysis) are equivalent to the costs incurred for juveniles prior to their independence (i.e. via parent-offspring disease transmission and reduced parental care by diseased parents), whereas the survival costs pre-independence certainly differ and may be more severe (see Results). Population-wide parameters were then obtained by weighting healthy and diseased vital-rate parameters according to disease prevalence (assuming no age differences in pox prevalence or disease risk; see \Lachish, #1427). We evaluated the potential population effect of Paridae pox at a range of population prevalence values (that encompassed, and slightly exceeded the range of observed prevalence at this population: 1%, 2%, 5%, 8%, 10%, 15%) [26].

## Results

### Impact of Pox on Great Tit Reproductive Performance

**Figure 1:**
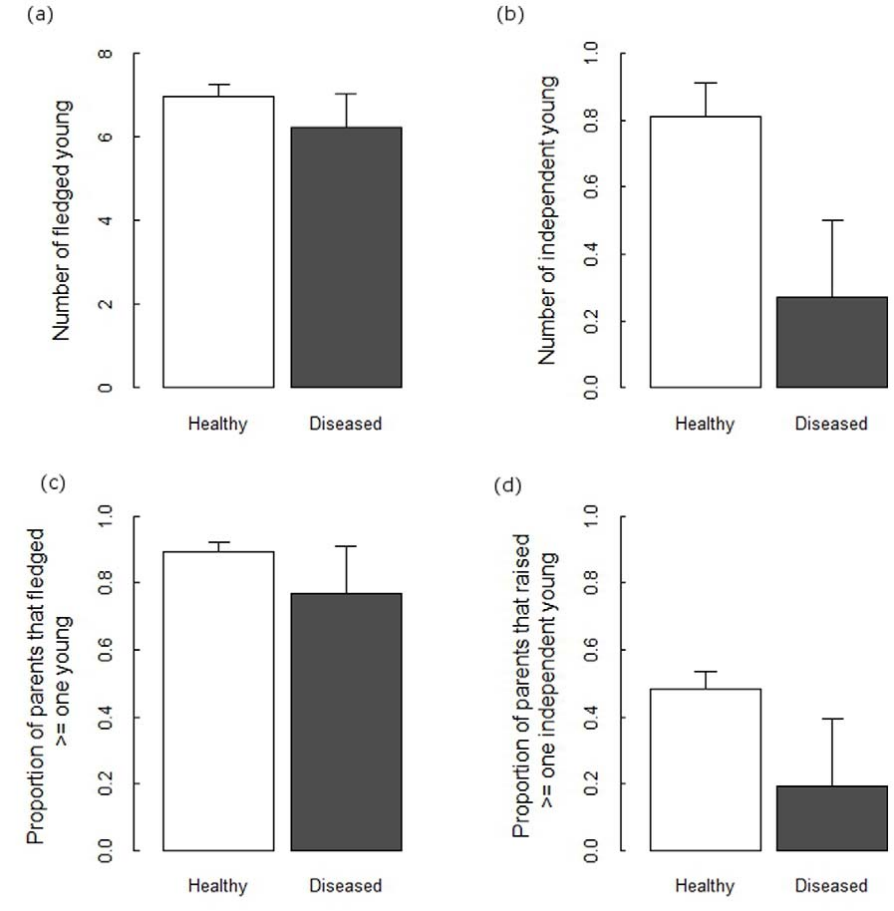
Reproductive performance measures for healthy and diseased great tit pairs at Wytham Woods. (a) average number of young fledged; (b) average number of independent young raised; (c) the proportion of pairs with at least one fledged young; (d) the proportion of pairs raising at least one independent young. Shown are the average estimates from the raw data (±95% CI).

Parental disease status (PDS) could be determined for 253 breeding attempts (of 316, 80%) in 2010 and 172 breeding attempts (of 234, 73.5%) in 2011, of which 20 and 6 (respectively) had at least one ‘diseased’ parent. The distribution of pox cases within great tit pairs did not differ from an expectation of independent association based on the prevalence of pox in the population (2010: χ^2^ = 0.30, df = 2, P = 0.86; 2011: χ^2^ = 0.07, df = 2, P = 0.96). Despite the relatively small number of ‘diseased’ pairs, results of generalised linear model selection revealed that the reproductive performance of ‘diseased’ pairs was significantly lower than healthy pairs for all reproductive measures, except clutch size and the number of young alive at day 15 (Table 2). Diseased pairs fledged fewer young on average than did healthy parents and were more likely than healthy pairs not to fledge any young at all (Table 2, Figure 1a & 1c). Stronger negative impacts were seen for analyses involving independent young (Table 2). Diseased pairs raised far fewer independent young than did healthy pairs and were far less likely than healthy pairs to produce any independent young (Figure 1b & d). Indeed healthy pairs were almost four times as likely as diseased parents to raise an offspring to independence (odds ratio 3.97, 95% CI 1.3–16.1, Fisher’s exact test P = 0.007). As we controlled for the number of young fledged by parents, this effect is independent of the negative impact of PDS on fledging success (Table 2). This is revealed by the fact that a far greater proportion of the fledglings raised by healthy parents were later seen as independent full-grown great tits (10.5%) compared with the fledglings raised by diseased parents (3.6%, χ^2^ = 7.56, df = 1, P = 0.006).

**Table 2:** The impacts of Paridae pox on reproductive performance of great tits.

Variable	Predictor	Estimate ± SE	*z*/*t*	P
(a) CS	**LD**	**−0.007±0.003**	**2.546**	**0.011**
	Density	**−**0.013±0.008	**−**1.725	0.084
	Year (2011)	0.021±0.033	0.651	0.515
	PDS (diseased)	0.048±0.067	0.717	0.474
	Year:PDS	**−**0.160±0.160	**−**0.997	0.319
	Year:LD	**−**0.006±0.007	**−**0.865	0.387
	Year:Density	0.007±0.016	0.460	0.645
(b) NYoung	**Clutch Size**	**0.097±0.011**	**8.748**	**<0.001**
	LD	**−**0.003±0.003	**−**1.156	0.248
	Density	**−**0.008±0.008	−1.020	0.308
	Year (2011)	0.030±0.035	0.863	0.388
	PDS (diseased)	0.051±0.070	0.737	0.461
	Year:LD	−0.007±0.007	−0.936	0.349
	Year:Density	0.002±0.016	0.095	0.925
	Year:PDS	−0.011±0.164	−0.064	0.949
(c) No. Fledged	**Nyoung**	**0.147±0.012**	**12.109**	**<0.001**
	**LD**	**0.007±0.003**	**2.145**	**0.033**
	**Density**	−**0.037±0.009**	**3.967**	**<0.001**
	**Year (2011)**	**0.209±0.094**	**2.234**	**0.026**
	**PDS (diseased)**	−**0.174±0.084**	**2.071**	**0.039**
	**Year:LD**	−**0.0219±0.008**	**2.697**	**0.007**
	Year:PDS	0.289±0.188	1.540	0.124
	Year:Density	0.019±0.019	0.999	0.319
(d) FledgedY/N	**Nyoung**	**0.276±0.083**	**3.324**	**<0.001**
	**LD**	**0.072±0.037**	**1.960**	**0.050**
	**Year (2011)**	**2.838±1.019**	**2.786**	**0.005**
	**PDS (diseased)**	−**1.192±0.522**	−**2.281**	**0.023**
	**Year:LD**	−**0.192±0.076**	−**2.518**	**0.012**
	Density	−0.106±0.073	−1.439	0.150
	Year:Density	0.053±0.163	0.324	0.746
	Year:PDS	−0.026±1.298	0.020	0.984
(e) NIndYoung	**NFledged**	**0.184±0.028**	**6.616**	**<0.001**
	**Density**	−**0.091±0.037**	−**2.477**	**0.013**
	**Year (2011)**	−**1.968±0.336**	−**5.854**	**<0.001**
	**PDS (diseased)**	−**1.113±0.413**	−**2.692**	**0.007**
	**Year:Density**	**0.214±0.073**	**2.923**	**0.003**
	LD	−0.018±0.012	−1.509	0.131
	Year:LD	−0.034±0.039	0.866	0.386
	Year:PDS	−13.97±781.48	−0.018	0.986
(f) IndYoungY/N	**NFledged**	**0.300±0.048**	**6.253**	**<0.001**
	**Density**	−**0.123±0.070**	−**1.760**	**0.079**
	**Year (2011)**	−**2.593±0.505**	**5.132**	**<0.001**
	**PDS (diseased)**	−**1.755±0.597**	**2.938**	**0.003**
	**Year:Density**	**0.269±0.111**	**2.424**	**0.015**
	LD	−0.032±0.021	1.543	0.124
	Year:LD	−0.031±0.056	0.559	0.577
	Year:PDS	−12.85±535.07	−0.024	0.981

Results of model selection for the effects of parental disease status (PDS), lay date (LD), year, and local breeding density (Density) on measures of reproductive performance in great tits: (a) clutch size (CS), (b) the number of young alive at day 15 (NYoung), (c) the number of young fledged (NFledged), (d) whether any young fledged (FledgedY/N), (e) the number of independent young produced (NIndYoung) and (f) whether parents produced any independent young (IndYoungY/N). Estimates of coefficients of the linear predictors (on the link scale, with standard errors, SE) are shown along with the relevant test statistics (z/t) and their significance (P). Statistics for significant terms (P<0.05, shown in bold) are from the minimal model, other values are at the point at which that factor left the model.

As expected, there were significant associations between reproductive performance measures and the biological and ecological covariates known to influence breeding output in great tits (lay date, local breeding density, and performance at early breeding stages), and also significant differences in reproductive output between the 2010 and 2011 breeding seasons (Table 2). Interactions between PDS and year were not significant however, suggesting that the effect of disease on reproductive performance was similar in the two years. Although our power to discern the magnitude of disease impacts on reproductive parameters in 2011 was low given that only six breeding attempts in this year were by diseased pairs, parameter estimates in this year were consistently lower for diseased pairs than for healthy pairs (see Table S3). Average brood weight at day 15 did not differ significantly between diseased and healthy parents (Mann Whitney test on brood weights standardised with years, W = 3777, P-value = 0.95).

Over the two breeding seasons we observed a total of six nests that contained between them 38 diseased chicks (ranging from 10% (1 of 10 chicks) to 100% (9 of 9 chicks) of all chicks in each nest). Nests with diseased chicks were significantly more likely to belong to diseased parents (odds ratio = 36.5, 95% CI 4.89–424.78, Fisher’s exact test, P<0.001).

### Impact of Pox on Great Tit Survival

A total of 1806 birds were captured during the eight capture occasions included in the multistate mark –recapture dataset. Of these individuals, 82 were diseased on at least one occasion, with 27 transitions observed from the healthy to the diseased state, and 10 transitions observed from the diseased to the healthy state. There was substantial temporal variation evident in recapture rates for healthy individuals (recapture rates were high during the breeding seasons and lower at other times, Table 3 & Table S2). There was strong support for an effect of disease state on recapture rates (Table 3), with lower recapture rates on average for diseased individuals (0.06, 95% CI 0.033–0.13) than for healthy individuals (average recapture rate = 0.285, 95% CI 0.194–0.424).

**Table 3:** The impact of Paridae pox on monthly recapture, survival and transitions rates of great tits.

Parameter Modelled	Model Structure			k	Deviance	QAICc	ΔQAICc	*w*
	Recapture	Survival	Transition					
*Most general model*	state*t	state*t	state*t	*46*	*4324.841*	*3420.535*	–	–
(a) Recapture rate (*p*)	H_t_+D†	H_t_+D	Inf_t_+Rec	32	4340.007	3403.309	0.000	0.802
	t			31	4346.315	3406.110	2.801	0.198
	state			26	4663.707	3640.025	236.716	0.000
(b) Survival rate (φ)	H_t_+D	state+age†	Inf_t_+Rec	27	4340.034	3393.09	0	0.423832
		state+age+H_sex_		28	4339.332	3395.102	2.0124	0.154955
		state*age		28	4339.749	3395.221	2.1314	0.146005
		H_age_+D		27	4343.429	3395.701	2.6115	0.114846
		state*age+H_sex_		29	4339.029	3396.408	3.3179	0.080672
		H+D_age_		27	4345.952	3397.642	4.552	0.043525
		age		26	4351.271	3399.69	6.6003	0.01563
		H_t_+age+D		33	4335.684	3402.037	8.9473	0.004834
		state*age+H_t_		34	4333.068	3402.079	8.9896	0.004733
		H_t+sex_+age+D		34	4334.033	3402.821	9.7318	0.003266
		H_t_+D		32	4340.007	3403.309	10.2192	0.002559
		H_t+sex_+D		33	4337.885	3403.73	10.6402	0.002073
		state		26	4356.706	3403.871	10.7814	0.001932
		state*age+H_t+sex_		35	4335.153	3405.739	12.6497	0.000759
		t		31	4347.642	3407.131	14.0409	0.000379
(c) Transition rate (ψ)	H_t_+D	state+age	Inf_t_+Rec†	27	4340.034	3393.09	0	0.994556
			Inf+Rec	21	4371.597	3405.134	12.0439	0.002412
			Inf_prev1_+Rec	22	4370.526	3406.345	13.2555	0.001316
			Inf_prev2_+Rec	22	4370.962	3406.68	13.5907	0.001113

Results of multistate mark–recapture analysis modelling the effect of pox on monthly recapture, survival and transitions rates of great tits. k = number of parameters; w = model weight.

† Most parsimonious model in each model selection step (retained for modelling subsequent steps).

‘*’ indicates interaction between variables; ‘+’ indicates additive effects; covariates in subscripts pertain only to the state/transition denoted; other covariates pertain to all states/transitions. See Table 1 for model notation.

**Figure 2:**
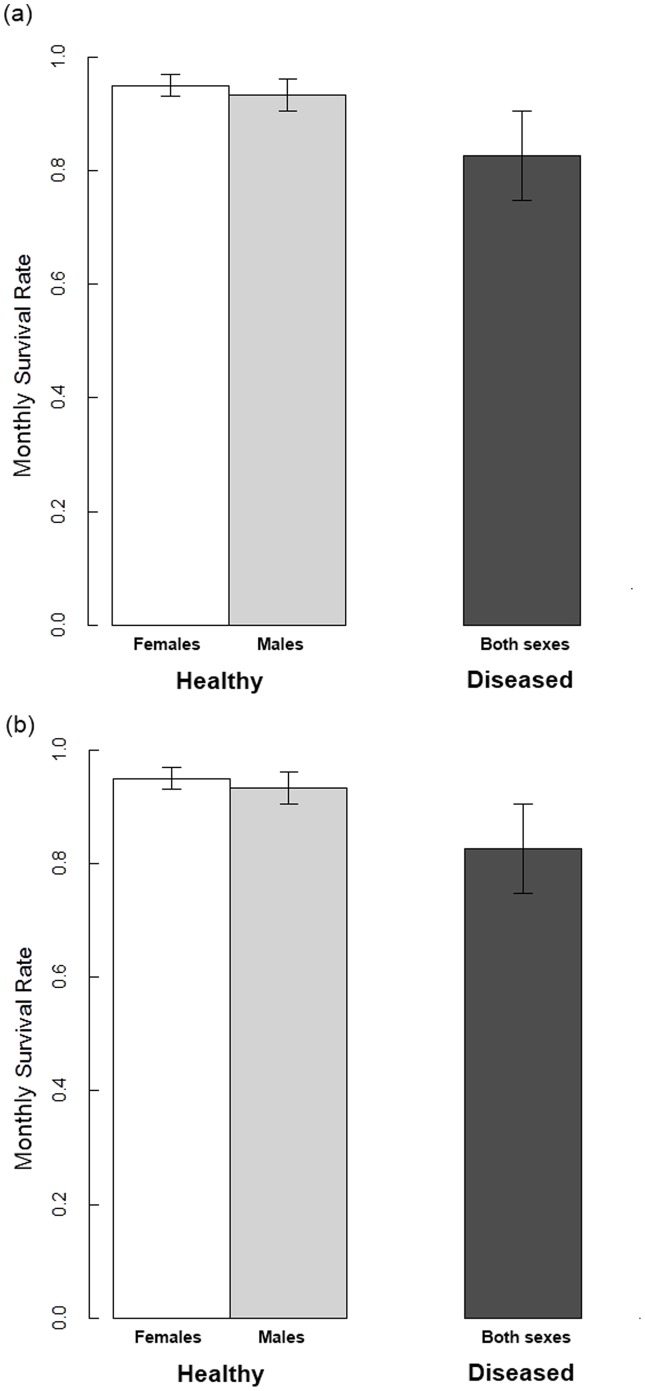
The impact of disease state on great tit monthly survival rates. Estimates shown are for (a) first years and (b) older individuals (white bars show healthy females; light grey bars show healthy males, dark grey bars show values for all diseased individuals). Estimates are model averaged mean monthly survival rates (±95% CI) obtained from multistate mark–recapture models.

Model selection revealed very strong support for an effect of disease state on survival rate, with all the top models in the candidate set containing the disease state effect (Table 3). Diseased individuals had a much lower survival rate than did healthy individuals (Figure 2; average effect size of disease on the logit scale (±95% CI) = −1.39 (−2.24 to −0.55)). In addition, we found strong support for an age difference in the survival rate of both healthy and diseased individuals (Table 3, only one of the top five models did not contain an age effect for diseased individuals). Model averaged estimates show that the proportional reduction in the monthly survival rate of diseased individuals was greater for juveniles than for adults, indicating that Paridae pox has greater impacts on the survival of younger individuals (Figure 2). This observation is further supported by the fact that none of the 38 diseased chicks were ever recaptured during the study (although 400 of the 3505 healthy chicks were recaptured at a later stage, χ^2^ = 4.0, df = 1, P = 0.047; generalised linear mixed model with year and disease status as fixed effects and family as a random effect), indicating a much higher mortality rate for diseased chicks compared to healthy chicks.

The best supported model of transitions rates allowed the infection rate to vary over time (Table 3). Surprisingly, models which attempted to relate this temporal variation in infection rates to the population prevalence of pox were not well supported (Table 3). Although the infection rates were high following capture occasions in which prevalence was high (see Table S1), this result suggests that the infection rate is determined by more than just the proportion of diseased individuals in the population (for example, by levels of vector activity, or the proportion of susceptible individuals in the population). Infection rates were inestimable (confidence intervals equal to zero) over the first and last time periods, as a result of the small number of transitions between healthy and diseased states observed at these times. Similarly, due to the paucity of observations of transitions from the diseased to the healthy state, the ‘recovery’ rate was inestimable in all models (confidence intervals for these estimates were always equal to zero).

### Impact of Pox on Population Growth Rate

**Figure 3:**
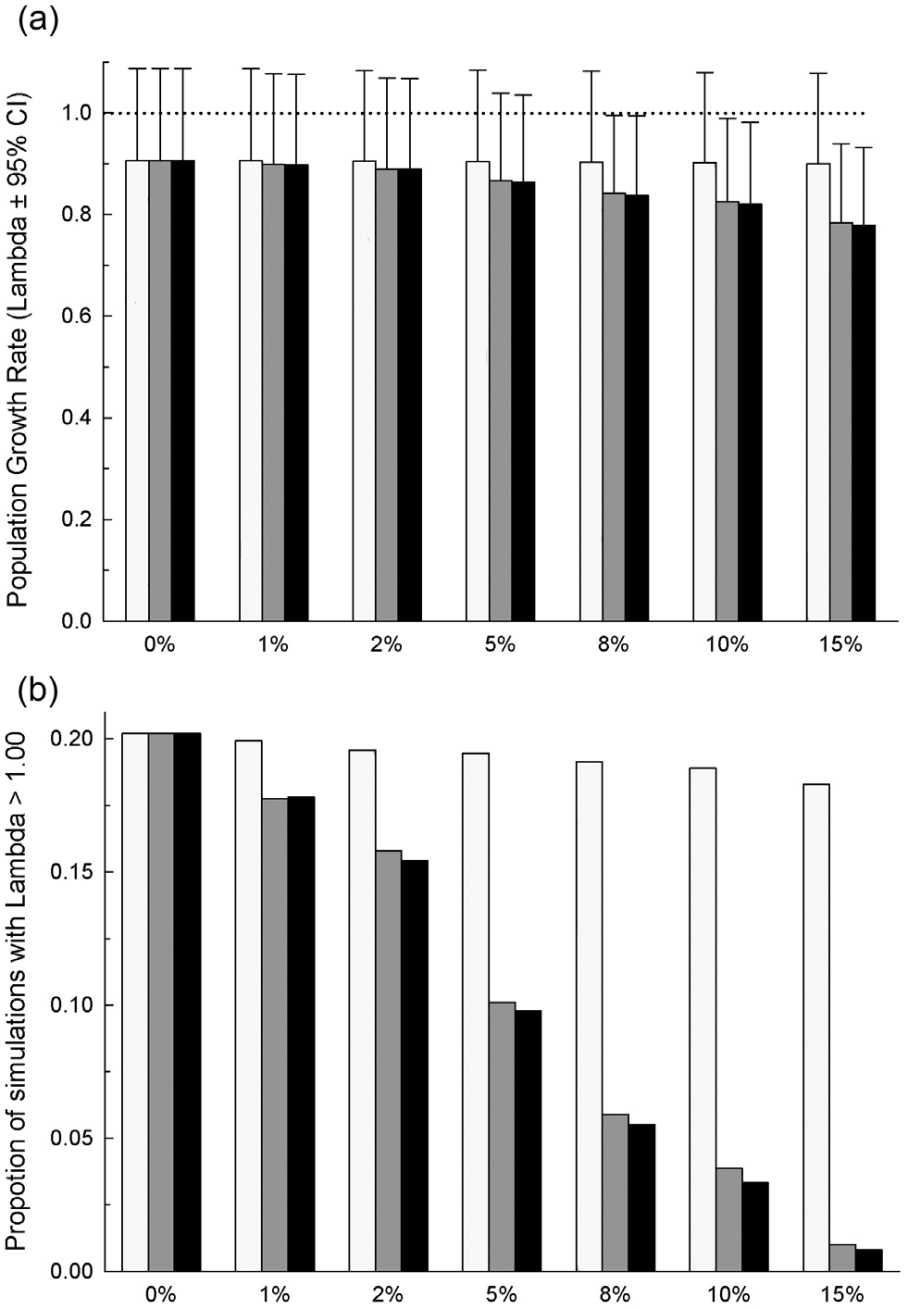
The influence of Paridae pox impacts and prevalence on great tit population growth rate. Results of the age-structured matrix model analysis to assess the influence of Paridae pox impacts and prevalence on the population growth rate (Lambda, λ) of the great tit population: (a) mean population growth rates (±95% CI) obtained from 10000 model runs in which pox impacts were incorporated on fertility rates alone (white bars), survival rates alone (grey bars), or on both vital rates together (black bars), and pox prevalence varied from 0% to 15%; (b) the proportion of each of the 10000 runs which gave λ ≥1.

The predicted average growth rate of the great tit population without pox was 0.91 (95% CI 0.78–1.09; Fig. 3a), with 20% of all model runs resulting in a population growth rate (λ) ≥1. That population growth was negative on average without pox (though 95% CI overlap one), is unlikely to be true in most years and is not consistent with demonstrated increases in great tit population size in recent years [45]. This apparent incongruity is most easily explained by our estimates of survival being biased low (which is particularly likely for juvenile survival, as this was estimated as “local recruitment of fledglings”, a measure that compounds survival, detectability and emigration). Comparing estimates of λ that included disease effects on fertility or survival alone, or on both vital rates combined, revealed that Paridae pox affects population growth rate primarily through reducing survival (Figure 3). As the effects of this disease are manifested predominantly through *per capita* losses (i.e. disease-associated mortality) the average growth rate of diseased populations decays almost linearly with disease prevalence (Figure 3). As expected, juvenile survival rate had a considerable influence on λ with sensitivity/elasticity analysis [38] revealing that λ is most sensitive to changes in this vital rate (see Table S2). Hence, the large variability in juvenile survival rates incorporated in model runs resulted in a wide range of possible population growth rates (λ) at all levels of pox prevalence (Figure 3a). Nevertheless, our results show that Paridae pox has the potential to reduce population growth rate, particularly when prevalence reaches 5% or higher, (which has been the average prevalence of pox in this population since disease emergence, Figure 3). In particular, when pox prevalence in the population is 8% or higher (the maximum recorded prevalence in this population to date was 9.7%) [26], population growth was negative in 95% of all model runs, and positive growth (i.e. λ>1) was only possible at extremely high juvenile survival rates (>18%; Figure 3).

## Discussion

### Individual Level Effects of Paridae Pox

Pathogens can negatively affect reproduction by reducing fertility rates of infected individuals, by compromising the nutritional status of infected individuals, by leading to behavioural changes that reduce the ability of infected individuals to care for their young [46], [47], [48], [49], or if the pathogen is transmitted from parent to offspring resulting in increased morbidity or mortality of the young [50], [51]. In this study, we have clearly shown that Paridae pox, a novel form of avian poxvirus disease, significantly reduces reproductive output in great tits, by reducing the ability of parents to fledge their young and, particularly, to rear their young to independence. The impacts of Paridae pox on the reproductive output of great tit pairs were evident only at these later stages of the breeding cycle and not during egg-laying, incubation, or earlier stages of chick feeding. Although we cannot discount the possibility that we lacked power to detect more minor disease impacts at earlier stages of reproduction, our results suggest that that the negative impact of Paridae pox on reproductive performance is manifested predominantly via impaired parental care. This may indicate a trade-off between immune function and the energetic demands associated with parental care, such that individuals investing heavily in an immune response have reduced ability to care for offspring [52]. Alternatively, pox lesions may interfere with vision and flight and therefore foraging ability or efficiency [21], with the greatest costs occurring at later breeding stages because the energetic demands of the young are greatest then [53], or because diseased individuals deteriorate (get sicker), or die, as time progresses. Transmission of poxvirus from diseased parents to their young was nevertheless possible in this study, and thus also likely contributes to the observed negative impact of Paridae pox on host reproductive output in this study (particularly as diseased chicks appear to suffer very high mortality rates).

Surprisingly, despite the obvious potential for a disease such as avian pox to compromise parental ability of affected individuals, few studies have attempted to quantify the impact of avian poxvirus on host reproductive output in the wild. Curry and Grant [54] reported lower hatching success in the Galapagos mockingbird (*Nesomimus parvulus*) in years with high pox prevalence, but the death of the incubating female due to disease was only one of several possible contributing factors. In contrast, Vanderwerf [55] reported no difference in the reproductive success of healthy elepaio (*Chasiempis sandwichensis*) pairs and pairs in which at least one bird had healed pox lesions, but no birds with active pox lesions were captured in that study. Interestingly, a study by Kleindorfer and Dudaniec [23] on Galapagos small ground finches (*Geospiza fuliginosa*), revealed that diseased males were less likely to be mated than were healthy males, indicating that the reproductive costs of poxvirus infection may arise from multiple physiological mechanisms.

A larger number of studies have investigated the effect of avian pox on host survival, reporting mixed results for the impacts of this disease both on juvenile survival (either fledging success or recruitment of young), and adult survival [18], [20], [56]. For example, Vargas [57] found that pox significantly increased the mortality rate of juvenile Galapagos mockingbirds, while Senar and Conroy [18] showed that the 15-day survival rate of diseased serins (*Serinus serinus,* the majority (84%) of which were juvenile birds) was reduced by almost 50% compared to healthy individuals. However, Young and Vanderwerf [58] found that avian pox did not reduce overall fledging success or fledgling rate of Laysan albatross (*Phoebastria immutabilis*) chicks, and observed the long-term survival of several diseased chicks. Similarly, avian pox has been shown to reduce adult survival rates in a variety of species [21], [59], [60], [61], but not in others [56], [62]. These variable results suggest that the impacts of avian pox on the survival of wild hosts are a result of species-specific differences in immune capacity and differences in the virulence of particular poxvirus strains [21], [63].

The results of our study show that Paridae pox is a virulent strain of avian poxvirus, which causes significant reductions in the survival of wild great tits. We found that disease impacts on survival were greater for younger birds (newly independent young of the year) than for older individuals, and appeared to be particularly severe for very young birds (none of the 38 chicks seen with pox lesions survived, compared with 11% of all healthy chicks). This suggests that differences in the immune capabilities of younger and older individuals influence the outcome of poxvirus infection in this species [64], [65]. Interestingly, differences in immune function do not appear to determine the susceptibility of younger and older individuals to this emergent poxvirus infection, as previous work has shown that disease risk does not vary by age in this population [26].

The magnitude of the disease impacts on monthly survival rates of great tits revealed by the multistate mark-recapture analysis implies that very few individuals will survive to breed again following poxvirus infection. Indeed, our estimates of the monthly mortality risk due to the disease equate to annual survival rates far lower (0.10) than the minimum values observed due to natural year to year variability in great tit survival rates at this study site over a 50 year period [39]. Nevertheless, a handful of apparent recoveries from pox lesions (N = 15) [26] have been seen in this population (albeit too few to allow a robust estimate of the recovery rate in our multistate model). The fact that Paridae pox is not invariably fatal for hosts suggests that a large component of the observed impacts of Paridae pox on survivorship is the result of diseased individuals being more vulnerable to other causes of mortality. In particular, as pox lesions can seriously impair an individual’s vision and flight [21], diseased individuals may become particularly vulnerable to predation. Indeed, as younger less-experienced birds are often common targets for predators [66], diseased youngsters might suffer a disproportionate cost of predation, providing an additional explanation for the more severe negative impacts of pox on survival of young birds seen in this study. In recent years there has been much interest in the potential influence of predation on host-parasite dynamics [67], with studies showing that selective predation on infected individuals can, under some circumstances, act to eliminate pathogens or prevent their establishment in populations [68], [69], but might facilitate pathogen persistence in situations where resistant individuals become less abundant [70]. The possibility that great tit hosts with Paridae pox experience lower survival rates as a result of greater predation pressures thus has important implications for understanding the long-term dynamics of this novel disease and warrants further investigation.

One potential limitation to the inferences regarding pox impacts drawn from our multistate mark-recapture analysis is thatsome infected birds in this study might have been misclassified as healthy (false positive classifications are considered very unlikely) [21]. Such misclassification would result in state-dependent survival rates that are biased low, and either greater or lesser disparity in the estimated state-dependent survival rates (depending on whether the misclassified individuals were more or less likely to be those that were later classified as diseased; see for example [18]). In this study, our survival rate estimates for healthy birds (model-averaged monthly survival rate for females of 0.95, equivalent to an annual survival rate of 0.54) were within the range of previous survival values recorded for healthy great tits in this population [39]. We therefore believe that the effect of misclassification on our analyses is minor (i.e. misclassification, if present, was not sufficient to depress the survival rate of birds in the healthy state).

Variation in the capture probabilities of diseased and healthy individuals can be an important source of bias in studies analysing changes in estimates of population prevalence, when such estimates rely on unadjusted capture frequencies [71]. The results of our multistate model revealed that recapture probabilities for diseased individuals were far lower than for healthy individuals, suggesting that naïve (field-based) estimates of Paridae pox prevalence in great tits will likely under-estimate true population prevalence. On the other hand, our analyses revealed no indication that the recapture probabilities of healthy and diseased birds varied between the two capture methods used in this study, nor as a function of the trapping effort per sampling period, and showed that recapture rates for diseased individuals did not vary strongly over time. This indicates that while naïve estimates of pox prevalence may be biased low, temporal variation in prevalence estimates will likely reflect changes in true population prevalence over time (and not simply variation in capture heterogeneity). However, as recapture probabilities of both healthy and diseased individuals outside the breeding season were very low it is likely that we lacked the statistical power to detect such variation. The autumn/winter capture protocol for the Wytham Woods long-term great tit monitoring program was designed to maximise the number of new individuals captured and marked prior to the breeding season, which resulted in artificially low recapture probabilities for marked individuals at these times. Implementation of a more systematic autumn/winter trapping protocol in the future would provide more accurate estimates of state-dependent recapture probabilities and the variation in these rates over time, and thus provide a more precise evaluation of the extent of bias present in naïve estimates of pox prevalence.

### Population Level Effects of Paridae Pox

While endemic avian poxvirus infections are often considered inconsequential to host populations [21], epidemics of avian poxvirus in naïve species or populations have been linked to substantial population declines [2], [22], [23]. The results of this study reveal that Paridae pox, a novel and particularly severe form of avian pox, has the potential to reduce population growth rates in a wild great tit population, and that this potential lies primarily through the negative impacts of the disease on host survival rates. While there is a degree of uncertainty in the exact estimates of age-specific vital rates for healthy and diseased individuals incorporated in the age-structured matrix models (which determine impacts on λ), we have clearly shown that Paridae pox causes significant reductions to host survival, with particularly large effects on juvenile survival, the vital rate to which λ is most sensitive. Hence, we believe that the possibility for a pox-induced reduction in the average growth rate of this population is a qualitatively robust finding. The magnitude of disease-induced reductions of population growth rate at low disease prevalence, however, was relatively minor. Moreover, it is evident that yearly variation in juvenile survival probabilities, which are largely driven by annual variability in environmental factors, for example resource abundance and winter severity [25], [40] will have a greater influence on population trajectories than does this disease. Nevertheless, our model revealed that significant disease-induced reductions in population growth rate could occur when pox prevalence exceeds 8%. While the prevalence of Paridae pox among great tits in this population displays seasonal peaks that can exceed 8%, the average prevalence in the population, and the most recent estimates of pox prevalence in the population, are lower than this (see Table S1) [26]. However as discussed above, given the low recapture probabilities of diseased individuals, naïve estimates of pox prevalence are likely to be biased low. At higher levels of pox prevalence we would expect to see disease-induced population decline in this population. Clearly then, obtaining accurate estimates of the population prevalence of Paridae pox in great tits is an urgent priority for our current research.

Nonetheless, due to their lower average growth rates, pox-affected populations can be expected to have greater difficulty in recovering from additional disturbances that further reduce their numbers. Hence, an additional significant conservation concern of this emerging Paridae pox epidemic is its potential to reduce the resilience of wild great tit populations to perturbation. While the status of great tits in the UK is secure, elsewhere in Europe (where increasing numbers of avian pox cases in wild tits also suggest that Paridae pox is an emerging disease, [72]; K. van Oers pers.comm) there is concern that great tit populations may be already stressed because of poor reproductive synchrony with their peak food supply due to a rapidly changing climate [73]. Under these conditions, the additional negative impact of Paridae pox on population growth rate can be expected to have greater conservation implications for population persistence. In addition, Paridae pox affects a wide range of species within the Paridae family [19] including populations of other more vulnerable, tit species (e.g. the marsh tit, *Parus palustris*, and the willow tit, *Parus montanus*), within which pox prevalence is less well-known [26], and might thus be more vulnerable to the negative impacts of disease [11].

Ultimately, a robust assessment of the impact of Paridae pox on the long-term population viability of wild tits will necessitate accurate estimates of disease prevalence as well as a thorough understanding of (i) the way this disease interacts with population density; (ii) the strength and seasonality of infection rates; (iii) whether diseased impacts on survival are additive (as is assumed by our population model) or compensatory (for example, if disease risk and impacts are greater for lower quality birds); (iv) whether demographic compensation occurs in response to disease impacts on survival (since, for example, reproductive success of great tits improves at lower population densities) [31]; and (v) variation in the extent and duration of any host immunity. While experimental infection studies would assist in understanding the course of infection in hosts, the majority of this information will only be obtained by the continual monitoring of disease prevalence in natural populations and the vital rates of diseased and healthy individuals. This study thus contributes to a growing body of work that highlights the value of long-term time-series data on disease progression and disease impacts on hosts, for accurately quantifying the threat that novel pathogens pose to wild populations and the dynamics of the host-pathogen relationship: information that is critical to guide appropriate management actions [50], [74].

## Supporting Information

Table S1 **Details of the capture occasions of great tits at Wytham Woods used in the multistate mark-recapture analysis to model recapture, survival and transition rates.**
(DOCX)

Table S2 **Vital rates for healthy and disease individuals used in constructing age-structured population models.**
(DOCX)

Table S3 **Estimates of the reproductive performance of healthy and diseased parents from each of the two breeding seasons.**
(DOCX)
