# Supplementary material for: Individual and Population-Level Impacts of an Emerging Poxvirus Disease in a Wild Population of Great Tits
Source: PLoS One. 2012 Nov 21;7(11):e48545. doi: 10.1371/journal.pone.0048545 (PMC3504048; doi:10.1371/journal.pone.0048545)
Supplement: Table S1 — Details of the capture occasions of great tits at Wytham Woods used in the multistate mark-recapture analysis to model recapture, survival and transition rates. (DOCX) [file pone.0048545.s001.docx]

**Table S1**: Details of the capture occasions of great tits at Wytham Woods used in the multistate mark-recapture analysis to model recapture, survival and transition rates. Shown are the number of individuals captured and the apparent prevalence of pox at each occasion. (* indicates breeding seasons when individuals were trapped at the nestbox; capture via mist netting occurred at all other times). Also shown are estimates from the best multistate model (see Results Table 3) of the recapture rates for healthy individuals and the infection rate (NB: recapture rates pertain to each particular capture session, while infection rates pertain to the interval between two capture sessions).

| **Capture Session** | **No. Individuals Captured** | **No. Diseased** | **Prevalence**  **(%)** | **Recapture rate** | **Infection rate** |
| --- | --- | --- | --- | --- | --- |
| May-09* | 512 | 1 | 0.195 | -- | -- |
| Nov-09 | 35 | 1 | 2.857 | 0.051 ± 0.026 | Not estimated |
| Feb-10 | 218 | 1 | 0.459 | 0.112 ± 0.022 | 0.046 ± 0.053 |
| May-10* | 554 | 20 | 3.617 | 0.775 ± 0.034 | 0.379 ± 0.064 |
| Nov-10 | 421 | 37 | 8.789 | 0.362 ± 0.063 | 0.375 ± 0.121 |
| Feb-11 | 93 | 6 | 6.452 | 0.063 ± 0.014 | 0.157 ± 0.112 |
| May-11* | 396 | 6 | 1.515 | 0.464 ± 0.063 | 0.103 ± 0.101 |
| Nov-11 | 323 | 11 | 3.406 | 0.138 ± 0.022 | Not estimated |
