# Supplementary material for: Individual and Population-Level Impacts of an Emerging Poxvirus Disease in a Wild Population of Great Tits
Source: PLoS One. 2012 Nov 21;7(11):e48545. doi: 10.1371/journal.pone.0048545 (PMC3504048; doi:10.1371/journal.pone.0048545)
Supplement: Table S2 — Vital rates for healthy and disease individuals used in constructing age-structured population models. (DOCX) [file pone.0048545.s002.docx]

**Table S2:** Vital rates for healthy and disease individuals used in constructing age-structured population models to assess the potential impact of Paridae pox on the growth rate (λ) of the great tit population. *The value for juvenile survival which was drawn for a normal distribution with a mean of 0.12 and standard deviation of 0.03. Also shown are the sensitivities and elasticities† of the components in the age-structured matrix constructed for a healthy population.

| **Parameter** | **Ageclass** | **Healthy individuals** | **Sensitivities** | **Elasticities** | **Diseased individuals** |
| --- | --- | --- | --- | --- | --- |
| 1. Fertility | 0-1 | 3.40 | 0.543 | 0.266 | 3.043 |
|  | 1-2 | 3.65 | 0.081 | 0.146 | 3.267 |
|  | 2-3 | 3.50 | 0.040 | 0.070 | 3.133 |
|  | 3-4 | 3.50 | 0.020 | 0.033 | 3.133 |
|  | 4-5 | 3.45 | 0.010 | 0.015 | 3.088 |
|  | 5+ | 2.19 | 0.005 | 0.002 | 1.960 |
| 1. Survival | 0-1^*^ | e.g. 0.149 | 1.774 | 0.277 | e.g. 0.001 |
|  | 1-2 | 0.49 | 0.246 | 0.120 | 0.039 |
|  | 2-3 | 0.50 | 0.101 | 0.051 | 0.040 |
|  | 3-4 | 0.48 | 0.036 | 0.017 | 0.038 |
|  | 4-5 | 0.45 | 0.005 | 0.002 | 0.036 |
|  | 5+ | 0.20 | 0.003 | 0.001 | 0.016 |

† sensitivities and elasticities were calculated as detailed in Caswell H (1989) Matrix population models: construction, analysis and interpretation. Massachusetts: Sinauer Associates Inc. 328 p
