# Supplementary material for: Individual and Population-Level Impacts of an Emerging Poxvirus Disease in a Wild Population of Great Tits
Source: PLoS One. 2012 Nov 21;7(11):e48545. doi: 10.1371/journal.pone.0048545 (PMC3504048; doi:10.1371/journal.pone.0048545)
Supplement: Table S3 — Estimates of the reproductive performance of healthy and diseased parents from each of the two breeding seasons. (DOCX) [file pone.0048545.s003.docx]

**Table S3:** Estimates (with standard errors) for healthy and diseased parents from each of the two breeding seasons (2010, with 253 breeding attempts of which 20 were by diseased pairs; 2011, with 172 breeding attempts of which 6 were by diseased pairs) for the four reproductive performance measures shown to be significantly associated with parental disease status: the number of young fledged: NFledged, the proportion of pairs that fledged at least one young: FledgedY/N, the number of independent young produced: NIndYoung, and the proportion of pairs that produced at least one independent young: IndYoungY/N (see Table 2 in manuscript for details).

| Parameter | Year | Healthy  Mean (± SE) | Diseased  Mean (± SE) |
| --- | --- | --- | --- |
| NFledged | 2010 | 6.87 ± 0.21 | 6.00 ± 0.89 |
|  | 2011 | 7.08 ± 0.23 | 6.83 ± 1.45 |
| FledgedY/N | 2010 | 0.87 ± 0.02 | 0.75 ± 0.09 |
|  | 2011 | 0.93 ± 0.02 | 0.83 ± 0.15 |
| NIndYoung | 2010 | 0.94 ± 0.07 | 0.30 ± 0.15 |
|  | 2011 | 0.58 ± 0.04 | 0.00 ± 0.00 |
| IndYoungY/N | 2010 | 0.54 ± 0.03 | 0.20 ± 0.09 |
|  | 2011 | 0.25 ± 0.03 | 0.00 ± 0.22 |
